# Anticholinergic Burden in People With Learning Disability and Mental Health Difficulties: A Baseline Audit

**DOI:** 10.1192/bjo.2024.625

**Published:** 2024-08-01

**Authors:** Sreeja Sahadevan, Melissa Hicks, Reena Tharian, Elizabeth Patteril, Regi Alexander

**Affiliations:** Little Plumstead Hospital, Hertfordshire Partnership University NHS Foundation Trust, Norwich, United Kingdom

## Abstract

**Aims:**

Background:

The cumulative effect of having one or more prescription drugs with anticholinergic properties is known as the Anticholinergic Burden (ACB). ACB can increase mortality and morbidity. Adults with learning disability are a high-risk group for this.

Aims:

To evaluate Anticholinergic Burden (ACB) for adults with learning disability and co-existing mental health conditions and make practice recommendations.

**Methods:**

A baseline audit was carried out over a period of 1 month on those treated within two specialist in-patient units in England. Routinely collected information including diagnosis, prescribed medication, clinical outcomes (Clinical Global Impression – CGI) and side-effect scales (ACB calculator, Anticholinergic Effect on Cognition – AEC, Liverpool University Neuroleptic Side-effect Rating Scale – LUNSERS, Glasgow Antipsychotic Side-effect Scale – GASS) were collated and analyzed using quantitative methods.

**Results:**

19 patients were included. Mean age was 37 years, 89% (n = 17) were male and 95% (n = 18) of white British ethnicity. The clinical diagnoses included 74% (n = 14) with mild learning disability, 68% (n = 13) with a major mental illness (psychosis or affective disorder), 53% (n = 10) with autism and 32 (n = 6) with a personality disorder. 89% (n = 17) were on antipsychotics, 63% (n = 12) on mood stabilizers, 58% (n = 11) on antimuscarinic drugs for antipsychotic side effects, 58% (n = 11) on anxiolytics, 32% (n = 6) on antidepressants and 89% (n = 17) on medication for physical conditions. Including pro re nata (prn) medication, the mean ACB calculator score was 6.68 and 4.21 on the AEC scale. On LUNSERS, the mean score was 23.13 (medium side effects), on GASS 8.87 (mild or absent side effects), on CGI efficacy index 6 (decided clinical improvement with side-effects not significantly interfering with functioning) and on CGI global improvement 2.37 (much improved).

**Conclusion:**

The ACB in people with learning disability and mental health problems is high. While an ACB calculator score of 3+ is described as associated with increased mortality and morbidity in the general population, the mean score in this sample was double that if prescribed prn medication was included. Medication prescribed for both mental and physical health reasons. Despite the high ACB, they show good clinical improvement and functioning regarding their mental health. The scores on other side effect scales are not exceedingly high either. There is a need for more research to set the specific practice standards regarding ACB measurement and monitoring in this group. Adequate health education for patients and staff should be a priority to maintain vigilance regarding ACB effects.

